# *IL-4* Gene Polymorphism May Contribute to an Increased Risk of Atopic Dermatitis in Children

**DOI:** 10.1155/2016/1021942

**Published:** 2016-04-24

**Authors:** Hong Shang, Xiu-Li Cao, Yu-Jie Wan, Jin Meng, Lu-Hong Guo

**Affiliations:** ^1^Department of Pediatrics, Jining No. 1 People's Hospital, Jining, Shandong 272111, China; ^2^Department of Emergency, Jining No. 1 People's Hospital, Jining, Shandong 272111, China; ^3^Department of Dermatology, Jining No. 1 People's Hospital, Jining, Shandong 272111, China

## Abstract

This study aimed to elucidate the associations between* interleukin-4 *(*IL-4*) single nucleotide polymorphisms (SNPs), 590C/T and 589C/T, serum IL-4 levels, and atopic dermatitis (AD) in children.* Methods*. A total of 82 children with AD were randomly selected as the case group and divided into mild group (15 cases), moderate group (46 cases), and severe group (21 cases). Additionally, 100 healthy children were selected as the control group. Genotype frequencies of IL-4 SNPs were detected by PCR-RFLP. Serum IL-4 levels were measured by ELISA.* Results*. Significant differences were shown in genotype distributions and allele frequencies of 589C/T and allele frequencies of 590C/T (all *P* < 0.05). Serum IL-4 levels in the mild, moderate, and severe groups were significantly higher than those in the control group; significant differences were found among these three groups with increased severity of AD. Serum IL-4 levels of heterozygote and mutant homozygote carriers in the mild, moderate, and severe groups were higher than wild homozygote carriers in those three groups and the control group (all *P* < 0.05).* Conclusion*. 590T and 589T alleles of* IL-4 *gene may be associated with high levels of serum IL-4, which may increase the risk of AD in children.

## 1. Introduction

Atopic dermatitis (AD), also known as atopic eczema, genetic allergic dermatitis, or constitutional prurigo, is the most common allergic inflammatory disease, which shows an apparent tendency of familial aggregation [1, 2]. AD is characterized by recurrence, pruritus, and inflammation, with clinically visible papules, erythema, exudation, erosion, incrustation, and lichenization changes, accompanying intense pruritus [3]. It has been reported that nearly 50% of children with AD develop symptoms within the first 6 months of life and approximately 85% of individuals with eczema have onset of symptoms by the age of 5 years [4, 5]. There are substantial differences in prevalence of AD between and within countries because of geographic distributions along with economic development. AD incidence in developed countries is now up to 10% to 20% [6, 7]. Until now, the pathogenesis of AD remains unclear, and previous studies on genetic epidemiology showed that AD was a polygenic disease [2, 8].

It has been confirmed that dysimmunity has been implicated in pathogenesis of AD, and differentiation imbalance of Th1/Th2 resulted in abnormal secretion of cytokines, which also played an important role in the development and progression of AD [9]. Interleukin-4 (IL-4) is a cytokine with various biological functions, which is secreted mainly by activated T cells and monocytes, basophilic granulocyte, and mast cells [10, 11]. In skin lesions of acute AD, activated Th2 cells would induce B cells to produce IgE by releasing cytokines such as IL-4 [9, 12]. As a characteristic cytokine of Th2 cell, IL-4 could promote the occurrence and development of inflammatory reactions characterized by Th2 [13]. A study showed that IL-4 gene polymorphism was associated with atopic asthma and allergic rhinitis both of which belong to complex inflammatory disorders [14], but there were few studies investigating the association between* IL-4* gene polymorphism and AD. Therefore, to verify this hypothesis, we carried out an association study on* IL-4* gene polymorphism and serum IL-4 levels with AD in children.

## 2. Materials and Methods

### 2.1. Subjects

Case-control design was adopted in this study. A total of 82 patients with AD, admitted to the Dermatological Department of Pediatrics in Jining No. 1 People's Hospital between January 2013 and May 2014, were randomly selected as the case group in this study. They were divided into mild group with score ≤ 20, moderate group with score between 20 and 50, and severe group with score ≥ 50 according to scoring atopic dermatitis (SCORAD) scoring [15]. A patient could be diagnosed as AD when he had pruritus history and three or more of the following characteristics: (1) history of flexor skin involvement, including fossa cubitalis, popliteal fossa, anterior talocrural region, or paracervical region (cheek was also included for children less than 10 years of age); (2) history of bronchial asthma or hay fever (or the occurrence of AD in children less than 4 years of age among first-degree relatives); (3) history of dry skin in the whole body; (4) visible dermatitis in the flexor side; and (5) AD occurrence before 2 years of age for patients of more than four years of age. In addition, 100 healthy children in this hospital were also selected as the control group.

### 2.2. Specimen Collection

Elbow venous blood (10 mL) was collected from each subject that fasted overnight for 10 to 12 h, and 3 mL of blood sample was transferred into vacuum tubes containing ethylenediamine tetraacetic acid (EDTA). Then, genomic DNA was extracted using whole blood genomic DNA extraction kit (Tiangen Biotech (Beijing) Co., Ltd.); the rest of the blood samples without EDTA were centrifuged for 10 min at 3000 rpm at room temperature to extract serum, which was then stored at −80°C.

### 2.3. Detection of* IL-4* 590C/T and 589C/T Single Nucleotide Polymorphisms (SNPs)

*IL-4* SNPs, 590C/T and 589C/T, were identified by polymerase chain reaction-restriction fragment length polymorphism (PCR-RFLP) method. The PCR primers were designed by Primer Premier 5.0 software and then synthesized by Shanghai Sangon Biotech Company. The primer sequences and lengths were shown in Table 1. The PCR reaction was performed in a total of 25 *μ*L containing 2.0 *μ*L DNA, 1.25 U TaqDNA amplifier enzymes (Sangon Biotech, SK2492), 2.5 *μ*L 10x PCR buffer, 2.0 *μ*L 2.5 mmol/L dNTP mix, and 20 pmol of each primer and sterilized in double distilled water. PCR reaction conditions were as follows: predegeneration at 94°C for 5 min, degeneration at 94°C for 30 s, annealing at 61°C for 45 s, extension at 72°C for 50 s, 35 cycles from degeneration to extension in total, and then extension at 72°C for 5 min. When the PCR reaction was terminated, 5 *μ*L of PCR products was identified by agarose electrophoresis. The PCR products were digested with MaeI and AvaII endonuclease in water at 37°C for 16 h, respectively, followed by 2% agarose gel electrophoresis, and genotyping results of 590C/T and 589C/T were obtained.

### 2.4. Detection of Serum IL-4 Levels

Enzyme linked immunosorbent assay (ELISA) was used to detect the IL-4 level in serum (reagent: Immundiagnostik AG Bensheim) according to manufacturer's instruction. Optical density value (OD value) of each well was measured at 450 nm wavelength. Linear regression equation of the standard curve was calculated using concentration and OD value of the standard substance, with O value of the specimen substituted into the equation to calculate the concentration of the corresponding specimen. All specimens were detected twice and the average was obtained, and the detection was in conformity with the quality control criteria of laboratory.

### 2.5. Statistical Method

Statistical analysis was conducted using SPSS19.0 software (SPSS Inc., IBM, Chicago, IL, USA). Continuous data was presented as mean ± standard deviation, and *t*-test or variance analysis was adopted for comparison between groups. Categorical data was presented with percentages or ratios, and Chi-square test was applied into comparison between groups. Hardy-Weinberg equilibrium was used to verify and evaluate the representativeness of the subjects in the control group. Differences in genotypes and allele frequency between the case group and the control group were presented as odds ratio (OR) with 95% confidence interval (CI). All *P* values were two-sided, and *P* < 0.05 was considered statistically significant.

## 3. Results

### 3.1. Baseline Characteristics

Among 82 children with AD, there were 56 males and 26 females, with a mean age of 5.09 ± 3.21 years, including 15 patients in the mild group, 46 patients in the moderate group, and 21 patients in the severe group. There was no significant difference in age, gender, and the severity of AD among these three groups (all *P* > 0.05) (Table 2). The control group consisted of 100 children who had normal findings on physical examination and also had surgical circumcision in our hospital, including 73 males and 27 females, with a mean age of 5.67 ± 3.38 years. No remarkable difference in age and gender was examined between the case group and the control group (both *P* > 0.05).

### 3.2. Distribution of* IL-4* SNPs, 590C/T and 589C/T

Genotype and allele frequencies of* IL-14* SNPs, 590C/T and 589C/T, were shown in Table 3. After examination by Hardy-Weinberg equilibrium method, all genotype and allele frequencies achieved genetic balance, with group representativeness. No statistical difference was found in 590C/T genotypes between the case group and the control group (both *P* > 0.05), while statistical differences existed in allele frequencies (both *P* < 0.05). Besides, there were significant differences in genotype and allele frequencies of 589C/T between the case group and the control group (both *P* < 0.05). T allele of 590C/T and T allele of 589C/T could increase the risk of AD (590C/T: OR = 1.78, 95% CI: 1.03–3.09, and *P* < 0.05; 589C/T: OR = 2.30, 95% CI: 1.25–4.22, and *P* < 0.01).

### 3.3. Association between the Severity of AD and* IL-14* SNPs, 590C/T and 589C/T

Genotype distributions and allele frequencies of 589C/T were exhibited among the control group, the mild group, the moderate group, and the severe group (all *P* < 0.05). However, no significant difference in genotype distributions of 589C/T was found between the control group and the mild group, the moderate group, and the severe group (all *P* > 0.05). As for allele frequencies of 589C/T, there was also no significant difference among the moderate group and the control group, the severe group and the control group, and the mild group and the moderate group (all *P* > 0.05). No significant difference was found in genotype distributions and allele frequencies of 590C/T among the mild group, the moderate group, the severe group, and the control group (all *P* > 0.05) (Table 4).

### 3.4. Comparison of Serum IL-4 Levels

Serum IL-4 levels in the case group and the control group were shown in Table 5. Compared with the control group, serum IL-4 levels of patients in the case group notably increased (85.34 ± 26.43 versus 38.66 ± 7.99, *t* = 5.228, *P* < 0.01). Serum IL-4 levels in the mild, moderate, and severe groups were all significantly higher than those in the control group (all *P* < 0.05). Statistical differences of serum IL-4 levels also existed among the mild group, moderate group, and the severe group with increased severity of AD (*F* = 52.08, *P* < 0.05).

### 3.5. Comparison of Serum IL-4 Levels of 590C/T and 589C/T

Serum IL-4 levels of AD patients carrying C/T + T/T of 590C/T and C/T + T/T of 589C/T were higher than those of the control group (all *P* < 0.05). In the case group, serum IL-4 levels of 590C/T C/T + T/T carriers were higher than those of CC carriers (both *P* < 0.05); also serum IL-4 levels of 589C/T C/T + T/T carriers were higher than those of the CC genotype carriers (both *P* < 0.05) (see Table 6).

### 3.6. Association between the Severity of AD and 590C/T and 589C/T

Compared with the control group, serum IL-4 levels of CT and TT (590C/T) carriers and TT (589C/T) carriers significantly rose in the mild group, moderate group, and severe group; serum IL-4 levels of CT (589C/T) carriers also rose notably in the mild group and the moderate group (all *P* < 0.05). Serum IL-4 levels of 590C/T CT carriers increased remarkably compared with those of CC carriers in the moderate group, and serum IL-4 levels of TT carriers were significantly higher than those of CT carriers in the moderate group and the severe group, respectively (all *P* < 0.05). Compared to 589C/T CT carriers, respectively, TT carriers in moderate group and the severe group had increased serum IL-4 levels (all *P* < 0.05). Serum IL-4 levels of AD patients carrying different genotypes of 590C/T and 589C/T increased with increased severity of AD (all *P* < 0.05) (Table 7).

## 4. Discussion

AD is a disease coregulated by multiple genes, approaches, and factors which attracted an increasing number of scholars to study its pathogenesis in recent years because of high incidence and lack of specific treatment plans. At present, it is reported that pathogenesis of AD mainly includes damaged epidermal barrier function, Th1/Th2 imbalance, and cytokines (IL-21, IL-25, and IL-33) effect [16]. Besides, previous study performed by Vafa et al. showed that the prevalence of* Plasmodium falciparum* infection was associated with the* IL-4* -590 T allele in Fulani ethnic group [17]. They also showed in another paper and proved the impact of the* IL-4* -590 C/T transition on the levels of* Plasmodium falciparum* specific IgE, IgG, and IgG subclasses and total IgE in two sympatric ethnic groups living in Mali [18] but there were few studies focusing on association between* IL-4* SNPs and the occurrence and development of AD.

Our study found that serum IL-4 level of patients in the case group increased significantly compared with that of the control group, and it would be elevated with increased severity of AD, which suggested that AD was closely related to the level of IL-4 in serum. IL-4 was the most specific cytokine of Th2 cell, and increased IL-4 showed that the Th balance of AD patients was shifting towards Th2. Namely, decreased expression of Th1 cytokine and increased expression of Th2 cytokine would lead to balance disorder and then acute AD skin lesions, and this elevation would increase with increasing severity of AD [19, 20]. In addition, IL-4 was the strongest IgE regulatory factor [21] which would combine with the interleukin-4 receptor (IL-4R) on cell surface, activate various nonreceptor protein tyrosine kinases (PTK) in cytoplasm to perform signal transduction, and then play various biological effects [10]. Moreover, IL-4 played a significant role in accelerating the synthesis of IgE, which could induce T cell proliferation and the homotype transformation from IgM to IgE [22]. Additionally, IL-4 also could promote the degranulation effect of mast cells, the differentiation from Th cells to Th2 cells, and the occurrence of IL-4 and IL-5 [11]. Differentiation imbalance of Th1/Th2 resulted in abnormal secretion of cytokines, which played an important part in the occurrence and development of AD [23].

In 1995, by using single stranded conformational polymorphisms (SSCP) and DNA sequencing methods, Rosenwasser et al. first reported that, in* IL-4* gene promoter region, the C→T mutation was detected in 590T base close to the glucocorticoid response element region, which was related to total IgE level since 590T would lead to increased IgE [24]. Kawashima et al. studied* IL-4* gene polymorphism among 88 core families with AD in Japan, and the result showed that carriers of T allele (590C/T) were predisposed to AD. The case-control study also suggested that there existed TT genotype association [25]. Combined with previous works, it was known that mutation of T allele of 590C/T and 589C/T would influence the expression of IL-4, eventually leading to the occurrence of AD [24–26]. Our study showed that mutant T allele prevailed in both the case group and the control group, and carriers of TT homozygote genotypes were much more predisposed to AD than carriers of CT heterozygote and CC homozygote genotypes, which further verified that 590C/T T allele and 589C/T T allele could enhance the risk of AD. T allele could increase the activity of* IL-4* gene promoter, and IL-4 played an important role in the improvement of IgE synthesis, suggesting that high frequencies of T allele would also increase the susceptibility of AD [22]. Collectively, this result hinted that IL-4 gene polymorphism was closely related to the occurrence and development of AD.

Consequently, serum IL-4 levels affect the occurrence and development of AD, and 590T and 589T alleles of* IL-4* gene are likely to be related to high level of IL-4 in serum, which may increase the risk of AD in children, providing new directions and ideas for the treatment of AD. However, study on the specific mechanism of AD remains unclear now, and more studies are required to conduct verification and analysis.

## Figures and Tables

**Table 1:** Primer sequences of *IL-4* single nucleotide polymorphisms, 590C/T and 589C/T.

	Primer sequences
590C/TFor	5′-GTAAGGACCTTATGGACC-3′
590C/TRev	5′-TACAAAGTTTCAGCATAGG-3′
589C/TFor	5′-TAAACTTGGGAGAACATGGT-3′
589C/TRev	5′-TGGGGAAAGATAGAGTAATA-3′

**Table 2:** Severity distribution of atopic dermatitis patients with different age and gender.

Variables	Severity of AD		
	Mild group (*n* = 15)	Moderate group (*n* = 46)	Severe group (*n* = 21)
Age (years)			
1–5	7	25	13
5–10	6	16	6
10–12	2	5	2
Gender			
M	7	16	3
F	8	30	18

Note: M, male; F, female; AD: atopic dermatitis.

**Table 3:** Allele and genotype frequency distribution of *IL-4* single nucleotide polymorphisms, 590C/T and 589C/T, in the case group and the control group.

	Case group (*n* = 82)	Control group (*n* = 100)	*P*	OR (95% CI)
590C/T				
CC	3	6		Ref.
CT	17	33		1.03 (0.23–4.64)
TT	62	61	0.111	3.03 (0.49–8.50)
CT + TT	79	94		1.68 (0.41–6.94)
C	23	45		Ref.
T	141	155	0.039	1.78 (1.03–3.09)
589C/T				
CC	1	3		Ref.
CT	15	36		1.25 (0.12–13.01)
TT	66	61	0.017	3.25 (0.33–32.06)
CT + TT	81	97		2.51 (0.256–24.56)
C	17	42		Ref.
T	147	158	0.007	2.30 (1.25–4.22)

Note: Ref., reference; OR, odd ratio; 95% CI, 95% confidence intervals.

**Table 4:** The severity of AD, allele frequencies, and genotype distributions of *IL-4* single nucleotide polymorphisms, 590C/T and 589C/T.

	Control group (*n* = 100)	Case group		
		Mild group (*n* = 15)	Moderate group (*n* = 46)	Severe group (*n* = 21)
590C/T				
CC	6	0	1	2
CT	33	4	10	3
TT	61	11	35	16
C	45	4	12	7
T	155	26	80	35
589C/T				
CC	3	0	0	1
CT	36	7	7	1
TT	61	8	39	19
C	42	7	7	3
T	158	23	85	39

**Table 5:** Comparison of serum IL-4 level between the case group and the control group.

Groups	*n*	IL-4 (pg/mL)
Control group	100	38.66 ± 7.99
Mild group	15	51.43 ± 8.94^a^
Moderate group	46	86.60 ± 17.79^ab^
Severe group	21	106.78 ± 26.69^abc^

Note: ^a^ *P* < 0.05, compared with the control group; ^b^ *P* < 0.05, compared with the mild group; ^c^ *P* < 0.05, compared with the moderate group.

**Table 6:** Effect of *IL-4* single nucleotide polymorphisms, 590C/T and 589C/T, on the serum IL-4 level in the case group and the control group.

	Genotypes	Case group	Control group	*t*	*P*
590C/T	CC	37.27 ± 9.12	36.41 ± 7.32	0.154	0.882
	CT	74.11 ± 19.28^a^	37.90 ± 6.26	7.545	0.000
	TT	90.82 ± 25.12^ab^	39.30 ± 4.16	15.930	0.000

589C/T	CC	89.26	34.02 ± 6.43	—	—
	CT	55.31 ± 15.82	36.52 ± 8.71	4.334	0.000
	TT	91.59 ± 24.26^b^	40.16 ± 7.37	16.420	0.000

Note: ^a^ *P* < 0.05, compared with wild homozygotes; ^b^ *P* < 0.05, compared with heterozygote.

**Table 7:** Association of the severity of AD with serum IL-4 level of *IL-4* single nucleotide polymorphisms, 590C/T and 589C/T.

	Genotypes	Control group	Mild group (*n* = 15)	Moderate group (*n* = 46)	Severe group (*n* = 21)
590C/T	CC	36.4 ± 7.3	—	37.2	37.3 ± 12.9
	CT	37.9 ± 6.2	49.3 ± 10.6^a^	77.2 ± 12.0^ab^	96.6 ± 9.9^abcde^
	TT	39.3 ± 8.9	52.2 ± 8.7^a^	90.7 ± 16.3^abe^	117.4 ± 13.1^abcde^

589C/T	CC	22.11 ± 2.6	—	—	89.26
	CT	31.52 ± 3.07	42.85 ± 2.88^a^	61.04 ± 10.72^ab^	95.53
	TT	43.69 ± 5.44	58.06 ± 6.71^ae^	91.19 ± 14.65^abe^	108.30 ± 27.66^abc^

Note: ^a^ *P* < 0.05, compared with the control group; ^b^ *P* < 0.05, compared with the mild group; ^c^ *P* < 0.05, compared with the moderate group; ^d^ *P* < 0.05, compared with the wild homozygote; ^e^ *P* < 0.05, compared with the heterozygote.
